# Mind–body reprogramming therapy: development, theoretical foundation, and clinical application for Long COVID

**DOI:** 10.3389/fpsyg.2026.1881081

**Published:** 2026-10-28

**Authors:** Henrik Børsting Jacobsen, Silje Endresen Reme

**Affiliations:** Department of Psychology, Faculty of Social Sciences, University of Oslo, Oslo, Norway

**Keywords:** brain–body framework, relational frame theory, chronic symptoms, interoceptive exposure, Long COVID, mind–body interventions, persistent physical symptoms, predictive processing

## Abstract

This paper presents the development and clinical rationale for Mind–Body Reprogramming Therapy (MBRT), a novel, theory-based intervention for patients with Long COVID experiencing persistent symptoms. Rooted in predictive processing, classical and operant conditioning, and Relational Frame Theory (RFT), MBRT targets maladaptive brain-based alarm responses believed to underlie symptoms such as fatigue, pain, and cognitive dysfunction. We argue that the chronicity of these alarms in patients reflects not only inferential biases and conditioned responses, but verbal relational networks that bind symptoms to identity, anticipated futures, and rule-governed avoidance, which become primary targets of treatment. The intervention combines personalized education, stress regulation techniques, and experiential learning delivered by an interdisciplinary team over a 7–10-day hybrid format. We describe the theoretical foundation of MBRT and outline its core components, including psychoeducation, values clarification, interoceptive exposure, and behavior change strategies. A detailed case study illustrates the therapeutic process and potential for recovery. We argue that persistent symptoms in Long COVID may reflect learned brain patterns rather than ongoing physical injury, and that targeted interdisciplinary interventions can help patients reverse these patterns. MBRT is currently under evaluation in a randomized controlled trial.

## 1 Introduction

Amid the global COVID-19 pandemic, a wave of persistent symptoms emerged, turning what was once a temporary illness into a prolonged struggle for many individuals. Biomedical research programmes are active and evolving (Peluso and Deeks, 2024), but so far no consensus mechanistic account has emerged, and no targeted drug treatment has been approved (Faghy et al., 2026). We drew on our research background in persistent physical symptoms and the stress response to develop Mind–Body Reprogramming Therapy (MBRT), a novel intervention designed to alleviate symptoms and support recovery.

The World Health Organization defines Long COVID as symptoms that persist or emerge 3 months after a COVID-19 infection and last for at least 2 months, with no other identifiable cause. The condition is associated with more than 200 symptoms, with fatigue, shortness of breath, and cognitive dysfunction being the most frequently reported (WHO, 2024). Additionally, individuals often experience post-exertional malaise, sleep disturbances, pain, loss of taste or smell, anxiety, and depression (Soriano et al., 2022).

Proposed explanations for the condition include theories that the virus, or remnants of it, persist in the body, leading to immune system overactivity or underactivity (Buonsenso et al., 2022). Other hypotheses suggest disturbances in the blood clotting system (Pretorius et al., 2021) or dysfunction in mitochondria (Appelman et al., 2024). Currently, these remain as hypotheses, as none have been substantiated by high-quality medical evidence derived from large-scale studies comparing individuals with Long COVID to unaffected control groups (Sneller et al., 2022). What we know, however, is that Long COVID manifests as clusters of persistent symptoms.

Looking more broadly, persistent physical symptoms are certainly not unique to COVID-19. They can occur after various bodily stressors, such as injuries or major surgeries (Crombie et al., 1998), and following other infections, such as mononucleosis (Hickie et al., 2006; Pedersen et al., 2019) and giardiasis (Wensaas et al., 2012). Certain biological abnormalities have been documented in patients with Long COVID, similar to what has been found in patients with other persistent symptoms, such as chronic pain (Schistad et al., 2017), CFS/ME (Strawbridge et al., 2019), and depression (Miller and Raison, 2016). However, these abnormalities do not necessarily imply causality. Instead, they may be reversible consequences of processes like prolonged stress (Munk et al., 2021; Wyller et al., 2009).

Indeed, observational studies have indicated that psychosocial factors are better predictors of Long COVID than biomedical factors (Kikkenborg Berg et al., 2022; Selvakumar et al., 2023; Stephenson et al., 2022), making a strong case for the potential benefits of treatments with a biopsychosocial approach. A recent systematic review aligns with this perspective, showing no evidence supporting the efficacy of medications, nutritional supplements, or medical devices as treatments for Long COVID. Instead, it provides moderate-certainty evidence supporting cognitive behavioral therapy and physical and mental health rehabilitation as effective approaches (Zeraatkar et al., 2024).

## 2 Theoretical foundations of MBRT

### 2.1 The brain and evolved survival mechanisms

No symptom can be experienced without the brain. Consequently, sensations such as fatigue and pain arise when specific brain networks are active (Wager et al., 2013). These networks can be activated by signals from the body, such as when an injury causes pain in a specific body part. However, they can also be activated by signals from the environment and other parts of the brain, meaning that a signal from the body is neither necessary nor sufficient to produce a symptom as experienced by the individual (Van den Bergh et al., 2017). The brain can also modulate the “volume” of signals received from the body. For instance, several studies have shown how specific changes occur in brain regions that govern attention (Malfliet et al., 2017), altering the significance of stimuli in patients with chronic pain conditions (Rehm et al., 2021).

Pain and fatigue serve as warnings indicating that the body’s internal stability is threatened. From an evolutionary standpoint, these symptoms protect and ensure survival. Because they signal a threat, they have extensive consequences, such as activating the body’s stress response to heighten alertness and promote behavioral changes to avoid actions that exacerbate the symptoms (Lasselin et al., 2018). This involuntary behavior can become persistent when the alarm system either becomes overly sensitive (Van den Bergh et al., 2021) or through conditioned learning (Munk et al., 2021; Pavlov, 1927; Vlaeyen and Crombez, 2020). It is now broadly recognized that some individuals may be more prone to persistent alarm responses due to a combination of genetic vulnerability, personality traits, and past life experiences (Van den Bergh et al., 2021).

When confronted with pain and fatigue, it is natural to resort to problem-solving. Typically, individuals employ strategies that have previously been effective. However, in the context of long-term symptoms, these strategies typically do not lead to symptom resolution. Paradoxically, some may even contribute to the persistence of symptoms (Hayes et al., 2006). For many individuals, a sense of hopelessness or helplessness arises when previously effective methods no longer work, leading to doubt about the efficacy of continued efforts.

### 2.2 Predictive processing

Predictive processing is a comprehensive framework for understanding brain function (Clark, 2023; Friston, 2003). It posits that the brain is constantly generating predictions about what to see, hear, feel, and experience, which helps us navigate effectively in the world. These predictions are hypotheses that are tested against sensory signals, both from the outside world and from our bodies (interoception). The goal is to minimize the difference between predictions and incoming sensations (i.e., minimizing prediction error).

Predictive processing theory has been applied to bodily symptoms, proposing that top-down processes influence not only how but also whether symptoms are experienced at all (Van den Bergh et al., 2017). Following these principles, experienced symptoms occur along a continuum of objectivity, meaning we can sometimes mistake noise (normal bodily processes) for symptoms such as pain, or even mistake inflammation or nociception as noise. These perceptual errors at lower levels of symptom perception are unconscious and beyond naïve dualistic mind–body models. They are better conceptualized as the joint product of conditioned learning and high-precision priors that propagate top-down through the predictive hierarchy (Lenaert et al., 2018), an account we extend in the next sections by specifying how, in human patients, those priors are shaped by language and self-referential meaning.

### 2.3 Classical and operant conditioning

Classical and operant conditioning are central elements of learning theory that explain how the brain learns to associate stimuli with each other and how behavior changes in response to consequences. Classical conditioning, introduced by Pavlov (1927), demonstrates how neutral stimuli, when repeatedly paired with an unconditioned stimulus, acquire the ability to elicit responses originally triggered by the unconditioned stimulus. This associative learning process operates largely below the level of conscious awareness and forms another pillar in MBRT. Skinner’s operant conditioning framework further explains how behaviors and symptom expressions can be maintained over time by reinforcement contingencies. For example, the avoidance of activities perceived as painful may be negatively reinforced by the temporary reduction of discomfort, even though this pattern ultimately limits recovery. Conditioning models, however, were largely developed with non-verbal organisms; they describe how stimuli acquire function through pairing and consequence, but they do not, on their own, explain how, in human patients, these functions migrate to stimuli that have never been directly paired, including words, anticipated events, and the self. Relational Frame Theory addresses this gap.

### 2.4 Relational frame theory

Relational Frame Theory (Hayes et al., 2001; Törneke, 2010) addresses what predictive processing and conditioning leave underdetermined in language users: the aversive function of an experience spreads to stimuli that have never been directly paired with malaise, including words, anticipated events, and the self. Two RFT processes carry the explanatory load here, derived relational responding and transformation of stimulus function.

Derived relational responding is the human capacity to relate any two events under arbitrarily applied contextual cues. A patient who reads online that exercise crashes Long COVID patients can frame exercise and crashing as causally related without ever having experienced such a crash; combinatorial entailment then draws walking, returning to work, and the body’s own arousal signals into the same relational frame. Conditioning gradients cannot readily account for these stimuli because no perceptual similarity links them; the connection is verbal. Transformation of stimulus function then propagates the aversive properties across the network (Dougher et al., 1994): once the future is causally framed with exercise, it acquires the function of a threat, although it has never been experienced. The stimulus changes function through its place in the network rather than through direct pairing (Vlaeyen and Crombez, 2020).

These mechanisms supply predictive processing models with high-precision, top-down priors (Edwards, 2023). A rehearsed self-referential frame such as “I am someone with Long COVID” can organize lower-level interoceptive predictions and bias ambiguous bodily sensations toward symptom inference. Two additional processes help explain phenomena that conditioning alone leaves underdetermined. Rule-governed behavior, particularly tracking and pliance, may account for avoidance that persists despite disconfirming experience; a rule such as “if I push, I will crash” instructs the system to follow the rule rather than the contingency, an established obstacle to exposure-based recovery (Hayes, 1989). Finally, deictic framing may contribute to self-as-context and, pathologically, fusion with the symptom: “I am exhausted” becomes constitutive of identity, raising the precision of the symptom prior.

These accounts complement rather than compete, each specifying a different level of the processes targeted in MBRT. Predictive processing describes the inferential machinery through which expectations shape symptom perception, making expectation updating a key target of psychoeducation and interoceptive exposure. Here, MBRT draws substantially on Pain Reprocessing Therapy (PRT), particularly its use of safety-oriented psychoeducation and somatic tracking to revise threat-based interpretations of bodily sensations (Ashar et al., 2022). Conditioning describes how symptom responses are acquired and maintained through learned associations, making extinction learning and behavioral experiments central treatment targets. RFT, in turn, specifies the verbal and contextual processes through which symptom-related functions can spread beyond directly conditioned stimuli to broader contexts, including the self and anticipated future. Interventions targeting inference or conditioned associations alone may therefore miss the relational scaffolding that helps maintain symptoms. MBRT addresses these relational networks through defusion, values clarification, deictic exercises, and augmental rules linking micro-choices to chosen life directions, while predictive processing and conditioning principles guide psychoeducation and experiential exposure that updates priors and extinguishes conditioned responses (Hayes et al., 2006; Jacobsen et al., 2020).

## 3 The MBRT intervention

MBRT is an interdisciplinary intervention delivered by a team of physicians alongside clinical psychologists or psychiatrists over a concentrated 7–10-day period (Figure 1). The hybrid format combines in-person sessions with digital modules and is grounded in the premise that brain-generated “alarms” create the expectation of injury or illness, repeatedly activating disease-specific neural networks that give rise to the symptoms that patients experience. MBRT aims to update the brain’s expectations, thereby turning off the alarm and reducing or eliminating symptoms.

**Figure 1:**
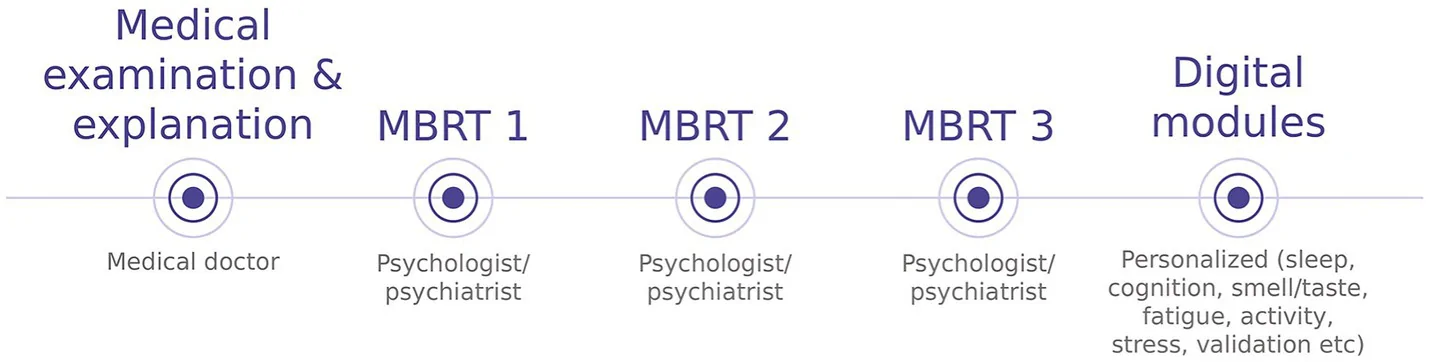
Overview of the mind–body reprogramming therapy (MBRT). The intervention is grounded in three theoretical pillars (predictive processing, classical and operant conditioning, and relational frame theory) and consists of two main components: an educational module and practical techniques targeting stress regulation and symptom alarm processing. MBRT is delivered in a hybrid format (face-to-face sessions combined with digital modules) by an interdisciplinary team over 7–10 days.

The specific techniques in MBRT are derived from evidence-based programs such as Pain Reprocessing Therapy (Ashar et al., 2022), Unlearn Your Pain (Holens et al., 2021), the Bergen 4-day treatment (Frisk et al., 2023), and our previous work with clinical hypnosis (Lind et al., 2021), Acceptance and Commitment Therapy (Fimland et al., 2014; Jacobsen et al., 2017), and stress research (Jacobsen et al., 2014; Munk et al., 2021).

We hypothesize that four sessions of MBRT, supplemented by digital follow-up, can lead to full recovery in adults with Long COVID. This is the pre-specified hypothesis under test, which is currently being evaluated in an ongoing randomized controlled trial. We also propose that MBRT may be effective for other persistent symptom disorders, though this has yet to be empirically tested.

Previous studies of mental and behavioral interventions similar to MBRT have not indicated an increased risk of adverse effects (Zeraatkar et al., 2024). Nevertheless, safety is closely monitored in the ongoing clinical trial, including routine assessment of any adverse events and symptom deterioration. Safety data are reported to and independently reviewed by an external Independent Data Monitoring Committee. The trial will also provide data on non-response and potential adverse effects.

### 3.1 Case illustration: applying MBRT

To illustrate the application of MBRT, we present an anonymized case, “Mary,” one of the first patients to undergo treatment. Mary was among the first patients to receive MBRT and, independently of the research team, chose to share her treatment experience publicly through popular media. We subsequently approached her to ask whether her case could be included in this article; she agreed. Her case was selected for its illustrative value in relation to the proposed mechanisms of MBRT and should not be interpreted as representative of treatment outcomes.

Mary’s story began in March 2022. During the pandemic, she was temporarily laid off from her job. When she became infected, she had just returned to work. The worst symptom Mary experienced was an intense, burning sensation in her throat that would not go away. She also struggled with brain fog, fatigue, difficulty breathing, and deep discouragement. She heard about the project and the opportunity to try MBRT.

#### 3.1.1 Session 1: personalized assessment, validation, and education

The first session begins by acknowledging the patient’s experience—reviewing the medical history, recent health events, and validating reported challenges such as fatigue, brain fog, and emotional distress. Relevant examination and test results are presented in accessible, non-technical language, offering reassurance about what these findings suggest regarding symptom origins and the potential for recovery.

Next, we introduce the concept of predictive processing, using everyday examples—such as optical illusions and the rubber hand illusion—to illustrate how the brain actively constructs sensory experiences. We explain how this predictive mechanism can sometimes misfire and lead to persistent symptoms in the absence of ongoing tissue damage.

Mary reported feeling both seen and heard during this consultation. She noted that she felt taken seriously for the first time in a long while and especially valued the explanation of how acute symptoms can become persistent through changes in brain processing.

The session concludes with the introduction of practical “neural reprogramming” exercises designed to begin shifting maladaptive predictions: a daily cold shower to help calibrate the body’s alarm system, morning self-affirmations aimed at reshaping expectation biases, and a reflective writing task in which the patient imagines and describes their ideal post-treatment self.

#### 3.1.2 Session 2: values clarification and micro-choices

In the second session, the patient meets with a psychologist or psychiatrist to explore and clarify core life values through structured exercises. These values serve as motivational anchors for future behavior change. The clinician then introduces the “microscope” technique—a detailed functional analysis of moments when symptoms tend to hijack behavior. For instance, the immediate urge to rest at the first hint of fatigue is examined in depth to reveal patterns of symptom-driven avoidance. By zooming in on these critical incidents, the patient begins to see how automatic responses, though understandable, may reinforce the very symptoms she is trying to escape. From an RFT perspective, the analysis also identifies verbally derived relations and rules (e.g., “if I push, I will crash”) through which otherwise neutral activities may acquire threat functions.

Building on these insights, the patient receives homework to deliberately engage in mildly symptom-provoking activities and practice making “micro-choices” that align with her identified values. These small but deliberate actions (e.g., taking a short walk instead of immediate rest) allow such rules to be tested against direct experience, bringing behavior into closer contact with actual contingencies while shifting focus from avoiding discomfort toward actively pursuing what matters.

#### 3.1.3 Session 3: defusion and activity as medicine

Session 3 begins with exercises designed to deactivate the brain’s “false alarms” through active reprogramming. This includes a guided interoceptive exposure that combines visualization, targeted suggestions, mindfulness, and the elicitation of positive emotions. Framed within a context of safety, these techniques help the brain reinterpret bodily signals as non-threatening—an essential condition, since mindful exploration only facilitates change when the patient feels safe, rather than frightened by their symptoms.

The session then introduces activity-based homework grounded in the concept of micro-choices. Whenever symptoms arise, the patient is encouraged to experiment with “doing something different”—for example, choosing a short walk instead of returning to bed. The goal is to replace avoidance patterns with meaningful engagement, reinforcing new predictive models of safety and capacity.

We emphasize that MBRT is a “doing” treatment, not just a “thinking” one, and liken consistent practice to taking prescribed medication: just as antibiotics are ineffective when doses are missed, the benefits of MBRT depend on regular and sustained participation.

Mary reflected on a key turning point: *“When I woke up feeling bad, I used to sit on the couch with coffee and TV, but that never helped. My therapist suggested I try something entirely different for 20 min — like having tea in the kitchen with the radio on. And most days, I found I no longer wanted to go back to bed.”*

From an RFT perspective, this experiment brought symptom-driven rules about the need to rest into contact with direct experience.

#### 3.1.4 Session 4: visualization and real-world exposure

The final session focuses on helping the patient integrate visualization and active reprogramming into everyday life. The session begins with a guided exercise: the patient vividly imagines a personally triggering situation and repeats the scene in their mind until it feels safe. We then explain how, much like in classical conditioning, the brain can form strong associations that turn harmless contexts into symptom cues.

Next, the patient practices reframing these triggers in real time. When encountering a previously discomforting scenario, they apply visualization and safety-priming techniques to actively “reprogram” the brain’s response—transforming perceived threat into an opportunity for regulation and mastery.

As Mary shared: *“I started taking longer walks in new places with my dog—something I rarely did before—and spent afternoons at the library reading about plants and endangered animals. By ‘doing something different’ in these small ways, my symptoms eased. Now I’m about 95 percent recovered, though I still feel overwhelmed in busy crowds on occasion.”* (Figure 2).

**Figure 2:**
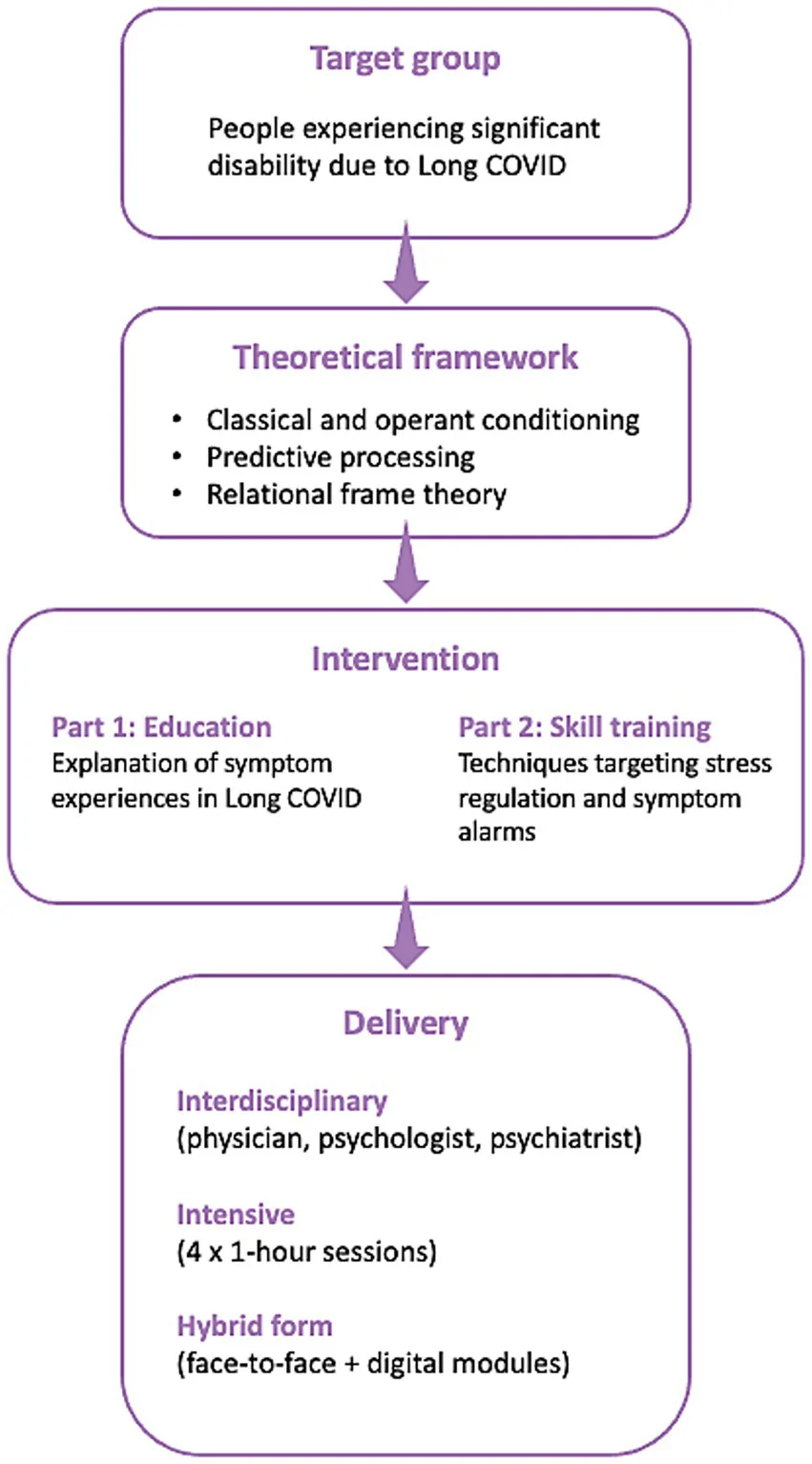
Structure of the MBRT treatment program. The program begins with an in-person medical examination and explanatory session delivered by a physician, followed by three structured MBRT sessions delivered by a psychologist or psychiatrist, with up to two additional sessions available if further repetition or clarification is needed. The initial medical session includes a detailed review of the patient’s medical history and recent health-related events, validation of the patient’s symptoms, and an explanation of persistent symptoms as potentially reflecting functional alterations in brain and bodily regulation. MBRT 1 focuses on functional analysis, validation, and exploration of core life values. MBRT 2 introduces specific exercises aimed at reducing inappropriate stress and threat responses, including visualization techniques adapted from somatic tracking principles in Pain Reprocessing Therapy. MBRT 3 consolidates these skills through repeated visualization and reprogramming exercises aimed at shifting responses to symptom triggers from threat and perceived unsafety toward safety. Participants also learn to apply these principles in daily life through graded exposure hierarchies. The in-person program is supplemented by personalized, self-paced digital modules addressing common symptoms and rehabilitation needs, including fatigue, cognitive difficulties, sleep, physical activity, stress regulation, and emotional awareness.

### 3.2 Digital continuation modules

To support patients in maintaining and deepening their recovery, we developed a series of self-paced digital modules that reinforce MBRT principles through targeted psychoeducation, guided exercises, and interactive tools. The modules are introduced at treatment onset and remain accessible throughout the three-month intervention period, allowing patients to use them before, during, and after the 7–10-day core program. Toward the end of the core program, clinicians may recommend specific modules based on individual symptoms and rehabilitation needs. Access is provided through the DIGNIO® interface.

The digital content comprises eight modules addressing common symptoms of Long COVID, including fatigue, “brain fog,” breathlessness, sleep problems, and altered smell or taste, as well as broader aspects of rehabilitation such as physical activity, emotional awareness, return to work, and support from family members. Each module combines information, sometimes delivered as brief online lectures, with suggested self-directed activities such as exposure exercises, meditation, self-hypnosis, and graded physical activity. Participants are encouraged to engage with modules relevant to their individual constellation of symptoms and functional challenges throughout the intervention period. A dedicated module for family members provides an introduction to MBRT principles and guidance on how to offer informed, validating support.

As Mary shared: *“I start each morning with a guided meditation in bed, using one of the audio files on the online platform. It really helps me set the tone for the day and calms my nervous system. I do not feel overwhelmed like I used to.”*

## 4 Discussion

Mary’s experience highlights how small, intentional “micro-choices” and the active disruption of ingrained habits can drive meaningful change. She entered the MBRT program with both motivation and an open mind, despite the ongoing and often polarized debate surrounding Long COVID treatments. Mary, like many others, was initially apprehensive that even light physical activity might exacerbate her symptoms. Under the structured guidance of MBRT, however, she gradually and safely reintroduced movement into her routine by systematically undoing the conditioned link between movement and symptom flare-ups.

### 4.1 Psychology is biology

Mary’s recovery underscores the need for a truly integrated understanding of Long COVID. We do not downplay biological factors, or the possibility of different mechanisms across symptom phenotypes, but we do emphasize how symptoms are generated by brain networks that respond to both internal bodily inputs (such as infection or injury) and external cues (such as sounds, smells, emotions, or memories) through associative learning (Bogaerts et al., 2023; Ishii et al., 2013; Van den Bergh et al., 2017). These conditioned “alarm” responses typically occur outside of our conscious awareness. Most people have experienced this: after food poisoning, simply seeing or smelling the culprit food can trigger nausea, despite the absence of actual danger. In humans this learning is amplified by relational networks that bind sensations to self-narratives, anticipated futures, and rules of conduct, which is why a single conditioned alarm can generalize far beyond its original context and resist new evidence.

Psychology and biology are inseparable: our thoughts and emotions produce tangible physiological changes. Chronic stress increases vulnerability to infections (Cohen et al., 1991); changes in mindset can alter vascular tone (Jamieson et al., 2012); and simply believing one is infected can trigger fever (Imataki and Uemura, 2021). Conversely, mind–body interventions show measurable benefits, such as improved glycemic control in type 2 diabetes (Sanogo et al., 2023) and observable changes in brain function in chronic back pain (Ashar et al., 2022).

### 4.2 Future directions

MBRT is currently being evaluated in MINIRICO (ClinicalTrials.gov NCT05703074), a 2 × 2 factorial randomized controlled trial including 310 adults with Long COVID at Akershus University Hospital. The trial compares MBRT with care as usual, alongside a double-blind comparison of nicotinamide riboside with placebo. The primary outcome is the General Health subscale of the 36-item Short-Form Health Survey at 3 months, with follow-up at 12 months. If shown to be effective, the concentrated format (7–10 days) could offer a scalable alternative to longer-term treatment programs. We also propose that the principles underlying MBRT—targeting learned brain alarm responses through psychoeducation, interoceptive exposure, and behavioral micro-choices—may be applicable to other persistent symptom conditions beyond Long COVID, such as fibromyalgia, chronic fatigue syndrome, and persistent post-surgical pain. These applications await empirical testing.

A key limitation of the current paper is that it presents a single case illustration rather than systematic outcome data. While Mary’s story illustrates the therapeutic process and its potential, generalization requires the controlled evidence that our ongoing trial is designed to provide. Additionally, the question of which patients are most likely to benefit from MBRT remains open and will be explored in future research.

## 5 Conclusion

Mind–Body Reprogramming Therapy is a novel, evidence-informed intervention for Long COVID that integrates comprehensive medical examination, targeted psychoeducation, predictive processing, principles of classical and operant conditioning, and Relational Frame Theory’s account of how verbal and self-referential networks sustain symptom function over time. MBRT helps patients to recalibrate and deactivate maladaptive “alarm” circuits in the brain, leading to a reduction in symptoms—and in some cases, full recovery. The theoretical framework and clinical application presented here provide the foundation for ongoing empirical evaluation.

## Data Availability

The original contributions presented in the study are included in the article/supplementary material, further inquiries can be directed to the corresponding author.
